# Association of social integration with cognitive function trajectories among Chinese older adults: evidence from the China health and retirement longitudinal study (CHARLS)

**DOI:** 10.3389/fnagi.2023.1322974

**Published:** 2024-01-11

**Authors:** Amu Mose, Yanhong Chen, Xiaoshuang Tan, Qingman Ren, Xiaohui Ren

**Affiliations:** ^1^Department of Health Behavior and Social Medicine, West China School of Public Health and West China Fourth Hospital, Sichuan University, Chengdu, China; ^2^The People's Hospital of Guangxi Zhuang Autonomous Region, Nanning, China

**Keywords:** cognitive function, social integration, trajectory, group-based trajectory model, older adults

## Abstract

**Background:**

The prevalence of cognitive impairment among older adults remains high. It has been proven that social integration is related to cognitive function. However, limited research has examined the association of social integration and its different dimensions with cognitive function trajectories of older adults.

**Methods:**

The data were from the China Health and Retirement Longitudinal Study (CHARLS) spanning 2013 (T1) to 2018 (T3). A total of 3,977 older adults were included in the final analysis. Cognitive function was measured with items from an adapted Chinese version of the Mini-Mini-Mental Mental State Examination (MMSE), while the measurement of social integration included three dimensions: economic integration, relational integration and community integration. A group-based trajectory model (GBTM) was used to identify cognitive trajectory groups among participants and an unordered multinomial logistic regression was employed to explore the association of baseline social integration and its three dimensions with cognitive function trajectories.

**Result:**

Three cognitive function trajectory groups were identified: low-decline group (24.1%), medium-decline group (44.2%) and high-stable group (31.7%). Comparing to the medium-decline trajectory group, older adults with higher social integration scores were more likely to be in the high-stable trajectory group (OR = 1.087, 95%CI: 1.007 ~ 1.174), while less likely to be in the low-decline group (OR = 0.806, 95%CI: 0.736 ~ 0.882). Among the different dimensions of social integration, older adults with higher community integration scores were more likely to be in the high-stable trajectory group (OR = 1.222, 95%CI: 1.026 ~ 1.456); Older adults with higher relational integration scores were less likely to be in the low-decline trajectory group (OR = 0.816, 95%CI: 0.734 ~ 0.906). The economic integration was not found to correlate with the cognitive function trajectories. Stratified analyses revealed that the association between community integration and cognitive trajectories was only significant among older adults aged 60 to 69, and the association between relational integration and cognitive trajectories was only significant among older adults who was agricultural household registration.

**Conclusion:**

The developmental trajectories of cognitive function among Chinese older adults are heterogeneous. Social integration is significantly related to the trajectories of cognitive function in Chinese older adults. Measures should be taken to promote social integration of Chinese older adults to reduce the decline of cognitive function.

## 1 Introduction

The world is witnessing an increase in both the absolute and relative proportions of the older adult population. The global older adult population is projected to reach 1.4 billion by 2030, and this trend of aging is expected to continue, with an anticipated escalation to 2.1 billion by 2050 (World Health Organization, 2022). Along with the aging population comes a multitude of health issues among the elderly. Cognitive impairment stands as a significant issue impacting the health of older adults.

Worldwide, the prevalence of cognitive impairment in older adults remains high. A survey conducted in the United States on 7,338 adults aged 65 and older revealed that the prevalence of cognitive impairment was as high as 19% (Aliberti et al., 2019). In the European region, cognitive function issues are even more prevalent, as evidenced by one study indicating that the prevalence of cognitive impairment among European older adults reached a staggering 30.2% (Zaganas et al., 2019). The prevalence of cognitive impairment among older adults in China is also concerning, having already reached 14.95% in 2019 (Liao et al., 2021).

Previous research has indicated that cognitive function in older adults develops dynamically with age and that there is heterogeneity in the trajectory of cognitive function among this population. In a 10-year study, Tran et al. (2021) tracked individuals aged 55 or older in Australia, revealing that 87.37% of them exhibited a high-normal cognitive function trajectory, with a small proportion of low-normal (11.33%) and declining (1.30%). Yuan et al. (2022) identified three distinct cognitive function trajectories within a cohort of 266,000 older adults in the United States: “Consistently Severe Cognitive Impairment,” “Consistently Moderate Cognitive Impairment” and “Consistently Intact/Mild Cognitive Impairment,” accounting for 35.5, 31.8, and 32.7% of all older adults, respectively. Similarly, Ye et al. (2022) identified three heterogeneous trajectories within the Chinese older adult population: high and declining (64. 24%), medium and increasing (19. 16%), and low and declining (16.60%). Evidently, the cognitive function trajectory within older adults does not consistently align, but illustrates a degree of heterogeneity. It is important to determine the developmental trajectories of cognitive function in older adults and explore the influencing factors for delaying the onset of cognitive impairment in this population. However, due to variations in research settings and participant characteristics, there are discrepancies in the number and patterns of developmental trajectories of cognitive function in older adults reported by different studies, necessitating further research to address this issue.

Durkheim initially introduced the concept of social integration, suggesting that boosting social integration could lower the suicide rate influenced by diverse social factors, but he did not provide a precise definition of social integration (Durkheim, 1992). There is currently no unified standard within the academic community for measuring social integration. However, there is broad agreement that social integration is not a single concept. Rather, it encompasses multiple dimensions. For instance, Yang (2015) posited that social integration should encompass economic integration, social adaptation, cultural identity, and psychological identification. Zhou (2012) proposed that social inclusion comprises five dimensions: economic integration, cultural adaptation, social adaptation, structural integration, and identity recognition. Similarly, Crawford (2004) asserted that social integration should involve political rights, economic engagement, equitable concern for social and cultural dimensions, and positive interpersonal bonds within families, friends, and communities. Through the review and analysis of numerous literature, Cordier et al. (2017) identified that the measurement of social inclusion primarily included three dimensions: connectedness, participation, and citizenship.

The current evidence shows a strong link between older adults’ social integration and cognitive function. Specifically, a higher level of social integration implies a better cognitive function. However, the dimensions of social integration focused on in these studies are not the same. Some studies have explored the relationship between relationships with friends (Bassuk et al., 1999; Pitkala et al., 2011), emotional support (Glymour et al., 2008), etc. and cognitive function in older adults, while most studies focused on the effects of social activity participation or social engagement on cognitive function (Zunzunegui et al., 2003; Tomioka et al., 2018; Sommerlad et al., 2019; Floud et al., 2021). The mechanisms linking social integration and cognitive functioning can be explained by the stress-buffering model based on social support theory, which suggests that social integration buffers the negative effects of stressful events on physical and mental health by increasing self-evaluation and decreasing subjective stress (Barrera Jr and Ainlay, 1983). In addition, social integration may have a neuroprotective effect on the brain according to biological mechanisms (Cacioppo and Hawkley, 2009).

Recently, a few studies have found that older adults with higher social integration status were more likely to experience positive cognitive function trajectories. In a study of Chinese older adults, Wang et al. (2022) found that older adults who participated in more social activities associated with a greater likelihood of experiencing a high-level and improved cognitive function trajectory. Similarly, one study conducted in Korea found that participants in the low-level and declining trajectory group were more likely to have low social participation (Son et al., 2023). Moreover, Ye et al. (2022) demonstrated that older adults with limited economic status and less physical activity were more likely to be categorized into the low initial level and rapid decline group as opposed to the high initial level and slow decline group. Nevertheless, such studies solely focused on specific dimensions of social integration, neglecting its multidimensional nature while examining its influence on the assignment of cognitive function development trajectories among elderly individuals.

In summary, existing studies have shown that there are different trajectories of cognitive function development within the older adult population, but there are differences in the type and number of trajectories derived in different studies, which need to be further investigated. Second, many studies have confirmed the correlation between social integration and cognitive functioning in older adults, but few studies have analyzed the relationship between social integration and cognitive function development trajectories, and even fewer studies have further explored the relationship between different dimensions of social integration and cognitive function developmental trajectories. Thus, this paper aims to identify the possible developmental trajectories of cognitive function in Chinese older adults, and how social integration, alongside its different dimensions, influences the ascription of cognitive function trajectories among Chinese older adults, to provide a basis for future research to apply social integration interventions to reduce the risk of cognitive impairment in older adults.

## 2 Methods

### 2.1 Data sources

The China Health and Retirement Longitudinal Study (CHARLS) is a large interdisciplinary longitudinal research cohort sponsored by the Beijing National Development Research Institute (NDI). CHARLS uses a stratified multistage probability-proportional random sampling strategy to survey middle-aged and older adults aged 45 years and older in 28 provinces (autonomous regions and municipalities directly under the central government) nationwide, including basic information about the respondents, household information, health status and function, health care and insurance, income, work and assets. The study’s national baseline survey was conducted in 2011, and follow-up surveys were conducted every 2–3 years thereafter, in 2013, 2015, 2018, and 2020 (Zhao et al., 2014).

Due to the large number of lost visits between 2011 and 2013, we used the data from CHARLS project in 2013 (T1), 2015 (T2) and 2018 (T3). We included participants based on the following criteria: (i) older adults aged ≥60 years; (ii) older adults who successfully returned to the survey in 2015 and 2018; and (iii) older adults with at least two measurements of the main variables, such as cognitive function and social integration.

A total of 8,723 older adults aged 60 years or older were surveyed in CHARLS 2013, of which a total of 6,521 older adults (74.76%) completed the three periods of follow-up. Excluding those with more than two periods of missing data on cognitive function (*n* = 2,519) and missing variables (*n* = 25), a total of 3,977 older adults were finally included in the analysis (The detail sample screening process was displayed in the Supplementary Figure 1).

### 2.2 Variable measurement

#### 2.2.1 Cognitive function

Cognitive function assessment tools in CHARLS, derived from an adapted Chinese version of the Mini-Mini-Mental Mental State Examination (MMSE), included five components: immediate memory, delayed memory, time orientation, calculation and attention, and drawing (McArdle et al., 2007; Luo et al., 2019). Interviewers presented 10 words to the participants for the immediate memory score and asked them to immediately recall as many as possible. Each correctly recalled word earned one point, with a score range of 0 to 10. Subsequently, after a period of time, participants were asked to recall the exact 10 words again for the delayed memory score with the same scoring system. The time orientation score was determined by the participants’ accurate responses to questions about the current year, month, date, day of the week, and season, with a score range of 0 to 5. The calculation and attention score were derived from participants subtracting 7 from 100 repeatedly for a maximum of five times, with one point awarded for each correct calculation and a score range of 0 to 5. For the drawing score, participants were instructed to reproduce a given image, earning one point for a successful reproduction, with a score range of 0 to 1. The total cognitive function score was the sum of these five components, with a possible range of 0 (worst) to 31 (best). In this paper, the Cronbach’s α coefficients for the years 2013, 2015, and 2018 were 0.80, 0.75, and 0.82, respectively.

#### 2.2.2 Social integration

Based on previous studies (Crawford, 2004; Zhou, 2012; Yang, 2015; Na et al., 2023), the measurement of social integration in this study included three dimensions: economic integration, relational integration and community integration.

##### 2.2.2.1 Economic integration

Previous research has shown that good economic status is the basis for older adults’ participation in other aspects of social integration (Huxley et al., 2012). Considering that most older adults have disengaged from the labor market, conventional economic integration indicators such as employment, job income, occupational prestige, and education are not applicable to this aging population. Therefore, in the present study, economic integration comprised two variables: housing structure and annual *per capita* household consumption level. The items for measuring the housing structure were as follows: “What is the structure of this building?” A score of 1 was assigned to indicate good housing conditions if “Reinforced concrete structure” was selected. A score of 0 was assigned to indicate average housing conditions if any of the following options were chosen: “Wood, bamboo, thatch structure,” “Felt house,” “Simple metal sheet house,” “Cave dwelling,” “Tent,” “Adobe house” or “Other structure.”

The household *per capita* annual consumption level reflected the economic status of older adults’ households, as it represents a stable and easily measurable expenditure level relative to income (Ren et al., 2019). Respondents were asked in the questionnaire, “How much money did your household spend in the past month/week? This includes expenditures on food, tobacco and alcohol, clothing, housing, daily necessities and services, transportation and communication, education, culture and entertainment, healthcare, and other goods and services. The total annual household consumption expenditure was calculated and then divided by the number of household members to obtain the *per capita* annual consumption expenditure (in Yuan) for the elderly. Based on the annual *per capita* consumption expenditures of rural and urban households in the corresponding years on the official website of the National Bureau of Statistics, the household *per capita* annual consumption level was finally transformed into a binary variable, categorized as “below average” and “average and above,” with respective assignments of 0 and 1.

The economic integration score was the average of the housing structure score and the *per capita* annual household consumption level score and contained three values, 0, 0.5 and 1, indicating low, medium and high, respectively.

##### 2.2.2.2 Relational integration

Relational integration encompassed two categories: family relationships and friend relationships. Family relationships are measured using the frequency of interactions with non-cohabiting children, while friend relationships were measured using the frequency of interactions with friends.

The frequency of interactions with non-cohabiting children was determined by the question, “When you are not living with your children, how often do you meet them or contact them through phone calls, text messages, letters, or emails?” The corresponding options were “Almost never,” “Once every two weeks or less,” “About once a week” and “Almost every day,” assigned values of 0, 1, 2, and 3, respectively. For childless older adults, we assigned a score of 0. Based on the CHARLS questionnaire, these two questions were skipped if an older adult did not have a non-resident child, and we treated them as missing values in our analysis (total of 12 older adults). If an older adult has multiple non-cohabiting children, the highest interaction frequency among the children was chosen (Lu et al., 2020).

The frequency of interactions with friends was determined by asking respondents, “In the past month, how often did you visit friends or engage with them?” The corresponding options were “Did not participate,” “Not often,” “About once a week” and “Almost every day,” assigned values of 0, 1, 2, and 3, respectively.

The relational integration score was the average of the scores obtained from interactions with non-cohabiting children and friend interactions. The score ranges from 0 to 3, with higher scores indicating better relational integration among the elderly.

##### 2.2.2.3 Community integration

Community integration was primarily measured through the following three items: “In the past month, playing Mahjong, chess, cards, or participating in community room activities,” “In the past month, dancing, exercising, practicing Qigong, or participating in park or other location activities” and “In the past month, participating in community or organization activities.” Each item corresponded to the options “Did not participate,” “Not often,” “About once a week,” and “Almost every day” with assigned values of 0, 1, 2, and 3, respectively.

The Social integration total score was the sum of the economic integration score, relational integration score, and community integration score. The score ranged from 0 to 7, with higher scores indicating better social integration among older adults.

#### 2.2.3 Covariates

Baseline socio-demographic characteristics (age, gender, marital status, educational level and household type) were considered for the adjusted analysis. Age is dichotomized into “Aged 60–69 years” and “Aged ≥70 years.” Marital status was divided into “Married” and “Not married.” The “Not married” category included those who are separated, divorced, widowed, or never married. Educational level was a three-category variable: “Illiterate,” “Primary school,” “Junior high school or above.” Household type was a dichotomous variable that included both “Agricultural” and “Non-agricultural” categories.

### 2.3 Statistical analysis

To describe the basic characteristics of the participants, frequencies were calculated for categorical variables and means and standard deviations were calculated for continuous variables. The group-based trajectory model (GBTM) was used to identify developmental trajectories of cognitive function in all the participants. GBTM is an exploratory technique used for isolating developmental trajectories within a population with the goal of identifying subgroups that follow distinct trajectories over time (Nagin, 2014). The best-fitting model was considered as the trajectory group with the highest probability, which was based on goodness-of-fit statistics via the Bayesian information criterion (BIC; the one whose absolute value of BIC is closest to zero is selected as the appropriate model; Su and Xiao, 2022). Finally, employing an unordered multinomial logistic regression, we analyzed the relationship between social integration and its three dimensions with cognitive function trajectories among Chinese older adults. To explore whether the association of social integration with cognitive trajectories varies across populations with different socio-demographic characteristics, we have further examined the interactions of age, gender, marital status, education level, and household type with social integration. The chi-square test was used to compare socio-demographic differences between the lost visit sample and the sample included in the analysis. We used the sampling weights from the baseline (2013) as weights for all our subsequent analyses. All data analyses were performed using Stata version 17.0. An association was deemed significant if the associated 2-sided value of p was less than 0.05.

## 3 Results

### 3.1 Characteristics of respondents in 2013 (T1)

Among the 3,977 older adults included in the analysis at baseline in 2013(T1), the proportion of individuals aged 60–69 was 74.22%, with males (58.62%) outnumbering females (41.38%). The majority of older adults were married, with a marriage rate of 84.90%. The educational level of the participants was relatively low, with primary school education being predominant, accounting for 51.61%. The proportion of older adults with agricultural household registration was 64.35%. The total social integration score was 2.14 ± 1.15 (Table 1).

**Table 1:** Basic characteristics of participants at 2013(T1).

Variables	Categories	N(%) or Mean ± SD
Age	60 ~ 69	2,952(74.22)
	≥70	1,025(25.78)
Gender	Male	2,331(58.62)
	Female	1,646(41.38)
Marital Status	Not married	601(15.10)
	Married	3,376(84.90)
Educational level	Illiterate	516(16.10)
	Primary school	1,653(51.61)
	Junior high school and above	1,034(32.29)
Household type	Agricultural	2,551(64.35)
	Non-agricultural	1,413(35.65)
Economic integration	Low	355(8.92)
	Medium	2,811(70.68)
	High	811(20.40)
Relational integration		1.26 ± 0.94
Community integration		0.31 ± 0.50
Total social Integration		2.14 ± 1.15

SD, standard deviation.

**Figure 1:**
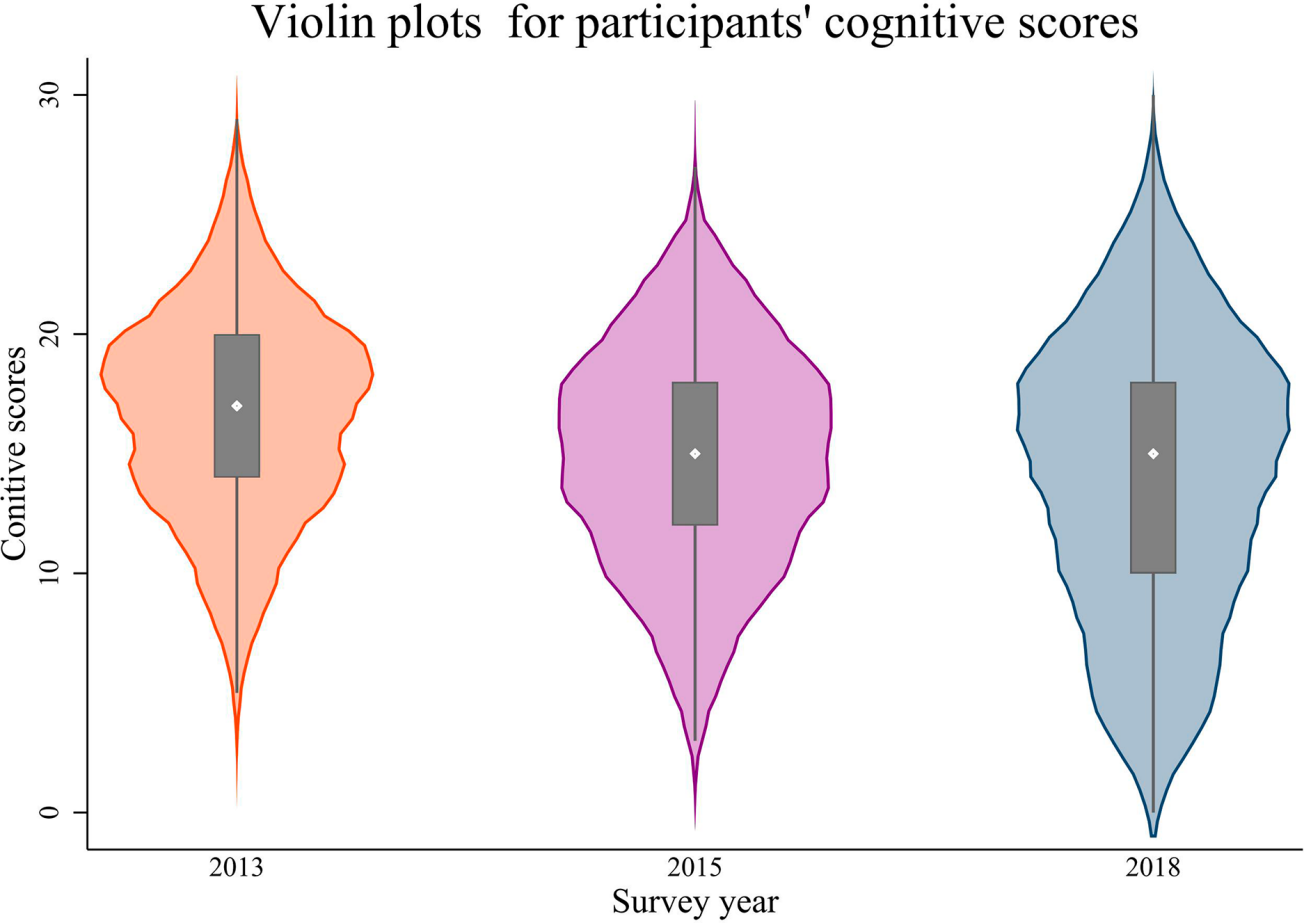
Violin plot of cognitive function scores for participants from 2013 to 2018.

Across 2013 to 2018, the cognitive function of the older adults was measured at 16.60 ± 4.44, 14.80 ± 4.73, and 14.02 ± 5.95, respectively. As depicted in Figure 1, it is evident that while the older adults’ cognitive function displays a declining trend, the internal variability is also increasing. This suggests the possibility of diverse cognitive function developmental trajectories within the Chinese older adult population.

### 3.2 The relationship between social integration and its three dimensions with cognitive function trajectories

**Figure 2:**
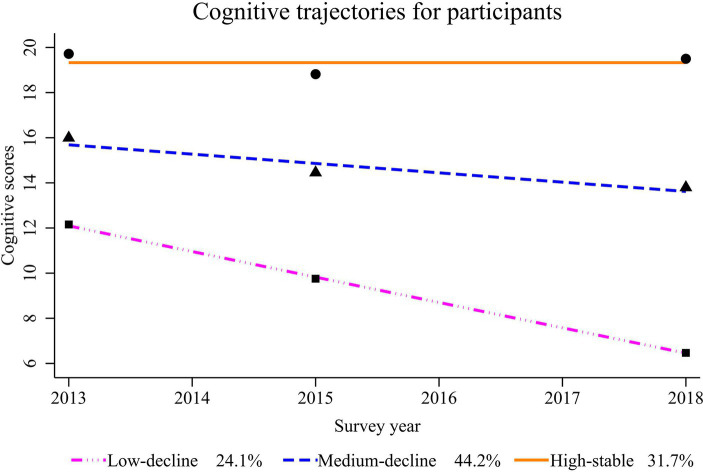
Potential cognitive function developmental trajectories for participants from 2013 to 2018.

Based on GBTM, an optimal model (BIC = −30074.83) was obtained (we tried several models and the parameters of the different models were shown in the Supplementary Table 1), the parameter estimates of the trajectory curves of the resulting model were shown in Table 2, and the morphology of the trajectories was shown in Figure 2. According to the best-fitting model, there was an overall declining trend in cognitive function among participants, with three distinct developmental trajectory groups identified: the low-decline group(24.1%), which experienced a rapid decline from a low starting level; the medium-decline group (44.2%), described as a slow decline from a medium starting level; and the high-stable group (31.7%), which had a high starting level and remained stable. The group with the lowest cognitive starting level (the low-decline group) experienced the most significant decline.

**Table 2:** Estimated results of cognitive function trajectory parameters for participants.

Trajectory group	Trajectory characteristics	Parameters	Estimated values	Standard error	t	*p*
1	Low-decline group	Intercept	2301.462	125.743	18.303	<0.001^***^
		Linear term	−1.137	0.062	−18.235	<0.001^***^
2	Medium-decline group	Intercept	847.383	155.595	5.446	<0.001^***^
		Linear term	−0.413	0.077	−5.347	<0.001^***^
3	High-stable group	Intercept	19.329	0.222	86.872	<0.001^***^

^*^indicates *p* < 0.05, ^**^indicates *p* < 0.01, and ^***^indicates *p* < 0.001.

Taking the medium-decline group as the reference group, we examined the association of the total social integration and its three dimensions with the cognitive function trajectories. The results indicated that older adults with higher total social integration scores were more likely to be in the high-stable trajectory group (OR = 1.087, 95%CI: 1.007 ~ 1.174), while less likely to be in the low-decline group (OR = 0.806, 95%CI: 0.736 ~ 0.882). In terms of the socio-demographic characteristics of participants, older adults who were older, male, not in marriage, had low levels of education, and were in agricultural household were more likely to be in the low-decline group (Table 3).

**Table 3:** Logistic regression analysis of the relationship between total social integration and cognitive function trajectories.

Variable	Low-decline group		High-stable group	
	OR (95%CI)	*p*	OR (95%CI)	*p*
Total social integration	0.806 (0.736,0.882)	<0.001^**^	1.087 (1.007,1.174)	0.033^*^
Sex (Ref. Male)				
Female	1.176 (0.955,1.448)	0.127	1.537 (1.271,1.858)	<0.001^***^
Age (Ref. 60 ~ 69)				
≥70	2.018 (1.614,2.522)	<0.001^***^	0.392 (0.312,0.492)	<0.001^***^
Marital Status (Ref. Not married)				
Married	0.598 (0.454,0.787)	<0.001^***^	0.928 (0.699,1.232)	0.607
Educational level (Ref. Illiterate)				
Primary school	0.343 (0.272,0.432)	<0.001^***^	3.429 (2.244,5.240)	<0.001^***^
Junior high school and above	0.107 (0.073,0.157)	<0.001^***^	9.684 (6.257,14.987)	<0.001^***^
Household type (Ref. Agricultural)				
Non-agricultural	0.389(0.294,0.515)	<0.001^***^	2.724(2.248,3.301)	<0.001^***^
Cons	2.655(1.752,4.023)	<0.001^***^	0.080(0.047,0.138)	<0.001^***^

OR, the odds ratio; CI, confidence interval; ^*^indicates *p* < 0.05, ^**^ indicates *p* < 0.01, and ^***^indicates *p* < 0.001.

Among the three dimensions of social integration, community integration and relational integration demonstrated a significant correlation with cognitive function trajectories. Specifically, older adults with higher community integration scores were more likely to be in the high-stable trajectory group (OR = 1.222, 95%CI: 1.026 ~ 1.456); Older adults with higher relational integration scores were less likely to be in the low-decline trajectory group (OR = 0.816, 95%CI: 0.604 ~ 0.906). The economic integration was not found to correlate with cognitive function trajectories in this study (Table 4).

**Table 4:** Logistic regression analysis of the relationship between three dimensions of social integration and cognitive function trajectories.

Variable	Low-decline group		High-stable group	
	OR (95%CI)	*p*	OR (95%CI)	*p*
Economic integration (Ref. Low)				
Medium	0.934 (0.672,1.297)	0.684	1.240 (0.865,1.778)	0.242
High	0.762 (0.524,1.110)	0.157	1.327 (0.893,1.972)	0.162
Relational integration	0.816 (0.734,0.906)	<0.001^***^	1.020 (0.927,1.122)	0.681
Community integration	0.778 (0.604,1.001)	0.051	1.222 (1.026,1.456)	0.024^*^
Sex (Ref. Male)				
Female	1.186 (0.963,1.460)	0.109	1.501 (1.241,1.815)	<0.001^***^
Age (Ref. 60 ~ 69)				
≥70	1.993 (1.593,2.493)	<0.001^***^	0.391 (0.312,0.490)	<0.001^***^
Marital Status (Ref. Not married)				
Married	0.615 (0.466,0.810)	0.001^**^	0.899 (0.676,1.195)	0.463
Educational level (Ref. Illiterate)				
Primary school	0.389 (0.277,0.440)	<0.001^***^	3.412 (2.231,5.219)	<0.001^***^
Junior high school and above	0.107 (0.073,0.158)	<0.001^***^	9.470 (6.111,14.675)	<0.001^***^
Household type (Ref. Agricultural)				
Non-agricultural	0391 (0.294,0.521)	<0.001^***^	2.657 (2.173,3.249)	<0.001^***^
Cons	2.501 (1.527,4.095)	<0.001^***^	0.075 (0.040,0.141)	<0.001^***^

OR, the odds ratio; CI, confidence interval; ^*^indicates *p* < 0.05, ^**^indicates *p* < 0.01, and ^***^indicates *p* < 0.001.

We further examined the interaction of relational integration and community integration with age, gender, marital status, education level, and household type. The results showed that the association between community integration and cognitive functioning trajectories varied by age, and the association between relational integration and cognitive functioning trajectories varied by household type (Supplementary Table 2). Stratified analyses revealed that the association between community integration and cognitive trajectories was significant among older adults aged 60 to 69, while no significant association was found among older adults aged 70 years and older. Within older adults aged 60 to 69, those with higher community integration scores were more likely to be in the high stability group (OR = 0.654, 95%CI: 0.466 ~ 0.919), while less likely to be in the low decline group (OR = 1.323, 95%CI: 1.075 ~ 1.628; Table 5). Similarly, the association between relational integration and cognitive trajectories was significant among older adults whose household type was agricultural, while no significant association was found among older adults whose household type was non-agricultural. Within older adults in agricultural household, those with higher relational integration scores were less likely to be in the low-decline group (OR = 0.822, 95%CI: 0.737 ~ 0.916; Table 6).

**Table 5:** Associations of social integration with cognitive trajectories stratified by age.

	Age(60 ~ 69)		Age(≥70)	
Variable	Low-decline group OR (95%CI)	High-stable group OR (95%CI)	Low-decline group OR (95%CI)	High-stable group OR (95%CI)
Relational integration	0.831 (0.733,0.943)^**^	1.035 (0.929,1.152)	0.816 (0.668,0.999)^*^	0.946 (0.760,1.178)
Community integration	0.654 (0.466,0.919)^**^	1.323 (1.075,1.628)^**^	0.977 (0.654,1.459)	1.024 (0.723,1.451)
Cons	4.015 (2.210,7.294)^***^	0.076 (0.037,0.154)^***^	1.952 (0.849,4.488)	0.010 (0.001,0.071)^***^

OR, the odds ratio; CI, confidence interval; ^*^indicates *p* < 0.05, ^**^indicates *p* < 0.01, and ^***^indicates *p* < 0.001. Adjusted by sex, marital status, educational level, household registration and economic integration.

**Table 6:** Associations of social integration with cognitive trajectories stratified by household type.

	Agricultural		Non-Agricultural	
Variable	Low-decline group OR (95%CI)	High-stable group OR (95%CI)	Low-decline group OR (95%CI)	High-stable group OR (95%CI)
Relational integration	0.822 (0.737,0.916)^***^	1.023 (0.906,1.155)	0.782 (0.569,1.073)	1.034 (0.878,1.219)
Community integration	0.776 (0.578,1.041)^*^	1.069 (0.799,1.431)	0.764 (0.461,1.268)	1.337 (1.047,1.706)^*^
Cons	2.371 (1.429,3.936)^**^	0.059 (0.027,0.130)^***^	0.855 (0.182,4.018)	0.256 (0.083,0.789)^*^

OR, the odds ratio; CI, confidence interval; ^*^indicates *p* < 0.05, ^**^indicates *p* < 0.01, and ^***^indicates *p* < 0.001. Adjusted by age, sex, marital status, educational level and economic integration.

## 4 Discussion

The present study used a group-based trajectory modeling (GBTM) approach to identify distinct trajectory patterns for Chinese older adults and explore the relationship between social integration and its three dimensions with possible trajectories of cognitive function among this population. Changes in cognitive function among Chinese older adults followed three distinct trajectories over 5 years. Social integration, particularly the community integration and relational integration, significantly correlated with cognitive function trajectories among Chinese older adults.

Our findings demonstrated that the cognitive function of older adults exhibited an overall declining trend with age, and the majority were in a medium-decline trajectory group. This trend is consistent with the results of Hayden’s study (Hayden et al., 2011). Moreover, we identified a low-decline trajectory, characterized by initially low cognitive function scores and the fastest rate of decline. Similar trajectories have been identified in the studies of Howrey et al. (2020) and Tran et al. (2021). Differently, we identified a high-stable trajectory within the older adults. Prior research has shown different developmental trajectories for older adults with high cognitive function starting level. For instance, Wu et al. (2022) has identified a developmental trajectory among older adults in Australia and the United States with high cognitive starting level, showing improvement over time. Conversely, two other studies have found a developmental trajectory of slow decline in older adults with a high cognitive starting level (Howrey et al., 2020; Tran et al., 2021). More targeted studies are needed in the future to explore the cognitive developmental trajectory of this particular group of individuals. Throughout the three trajectory groups, the group with the lowest cognitive function starting level experienced the most significant decline, consistent with the findings of Ma et al. (2022).

We measured the social integration of older adults in terms of economic integration, relational integration, and community integration in this paper. We found that social integration, particularly the community integration and relational integration, significantly associated with the trajectories of cognitive function among Chinese older adults. Specifically, older adults with higher social integration scores were more likely to be in the high-stable trajectory group, while the likelihood of being in the low-decline is reduced. Similar to the findings of this paper, a study conducted in the United States showed that community integration was associated with higher baseline cognitive functioning scores, with similar results across older adults of different ethnicities (Calmasini et al., 2022). As can be seen, although the measure of social integration in this paper differs from previous studies conducted in other regions (Secker et al., 2009; Mezey et al., 2013), which have also incorporated variables such as citizenship, participation in religious activities, the resulting relationships between social integration and cognitive functioning trajectories are similar.

The association between community integration and cognitive function has been confirmed by many studies (Tomioka et al., 2018; Floud et al., 2021). However, it was noteworthy that the association between community integration and cognitive trajectories was only significant among Chinese older adults aged 60 to 69 years in this paper. Community integration may have positive effects on older adults’ cognitive well-being through several pathways. Firstly, the process of community integration necessitates social interaction with peers, which inherently involves cognitive demands such as information reception and expression, event recall, and problem-solving. These activities could directly benefit for neural activity in the brain, potentially increasing brain capacity in the older adults (Zhao and Pan, 2018), or even counteracting hippocampal decline (Guo et al., 2018). The community integration in the present study involves diverse group social activities, including physical, cultural, intellectual, and other group activities. Participation in these community activities requires the engagement of cognitive or physical functions in older adults. For instance, physical exercises can enhance physiological functions in the elderly, stimulating cognitive functions related to spatial orientation and time perception (Zhao et al., 2020). Activities like mahjong and card games stimulate various cognitive functions, including memory and strategic thinking (Zhou et al., 2020). Moreover, many other community group activities have social attributes, and social support gained from these activities can contribute to improving cognitive function (Liu et al., 2019). Secondly, the social interactions and subsequent social support generated through the process of community integration might serve as a buffer against stress. This buffering effect involves diverting the older adults’ attention from stressful events, reducing the subjective evaluation of stress severity, enhancing coping mechanisms for stressful events, and consequently mitigating the detrimental impact of highly stressful events on both physical and cognitive functions (Glymour et al., 2008). The association between community integration and cognitive trajectories was not significant among Chinese older adults aged 70 years and older in this paper. This may be due to the fact that older adults over the age of 70 are less able to participate in community activities due to poorer physical functioning (Demura et al., 2003).

We found that the relational integration was associated with trajectories of cognitive functioning. However, this relationship was only significant among older adults with agricultural household registration. One of the reasons could be that older adults in agricultural households were more closely related to their relatives and friends (Liu and Wang, 2017), which was more significantly associated with their mental health. Previous studies (Nie et al., 2021; Meister and Zahodne, 2022) have shown that maintaining better connections with relatives and friends was positively associated with cognitive functioning in older adults, similar to the results of the present study. According to the social influence theory, relational integration might have a health education function, through interactions with relatives or friends, older adults could acquire health-related information, observe healthy behaviors demonstrated by others, or receive encouragement for healthy practices. This could lead to the adoption of habits conducive to health and protection of cognitive function (Thoits, 2011).

Differing from the findings of prior studies, this study did not discover a significant correlation between older adults’ economic integration and cognitive function trajectories. Vicerra’s study on the first wave of the Longitudinal Study on Aging and Health in the Philippines, which included 5,209 adults aged at least 60 years, found that greater wealth and income were associated with a slower decline in cognitive performance (Vicerra and Estanislao, 2023). Similarly, Gao et al. (2020) has indicated that poverty has a direct negative impact on cognitive function. One potential reason for this discrepancy is that approximately 75% of the surveyed elderly individuals were from rural areas. This relatively uniform socio-economic status among rural elderly participants might contribute to the lack of the observed correlation between economic integration and cognitive function (Gao, 2023). As a suggestion for future research, it would be valuable to investigate populations that have experienced long-term wealth disparities to better analyze the relationship between economic integration and cognitive function.

These findings can help us understand the developmental characteristics of cognitive function in Chinese older adults and develop targeted measures to slow down cognitive decline in Chinese older adults from the perspective of social integration. Older adults who were older, male, not in marriage, had low levels of education, and were in agricultural household may be the primary targets for enhancing cognitive function. Health professionals and researchers can use measures such as promoting participation in community activities and maintaining good contact with relatives and friends to slow cognitive decline in older adults. In addition, it is suggested that when using social integration intervention to reduce or slow down cognitive decline in the older adults, targeted measures should be developed based on the characteristics of the this population.

This paper may have several potential limitations: Firstly, due to the broad and multifaceted nature of the social integration concept, compounded by the absence of a standardized measurement scale and the constraints inherent in the existing CHARLS database, potential biases in the measurement of social integration might be present in this study. Secondly, although we found that the group with a better social integration exhibited a better cognitive trajectory, the causal relationship and mechanism underlying this transition remain unknown and warrant further research. Thirdly, We excluded a high number of participants lost visits or missing key variables, and there were significant differences in age, gender, marital status, and education level between those excluded and those included in the analysis (Supplementary Table 3). This may underestimate the proportion of low-decline groups and impact the effect size and generalizability of our findings. However, since CHARLS covers almost all regions of China and are nationally representative, the relationship between social integration and cognitive trajectories identified in this paper can still provide a reliable basis for further research.

## 5 Conclusion

Changes in cognitive function among Chinese older adults followed three distinct trajectories. Social integration, particularly the dimensions of community integration and relational integration, significantly correlates the trajectory of cognitive development in the Chinese older adults. It is recommended to pay attention to the older adults with low cognitive starting level and implement relevant measures to promote social integration among Chinese older adults to enhance cognitive health in later life. On the one hand, communities should frequently host a range of diverse activities to attract active participation from older adults, fostering their integration within the community. On the other hand, promote the establishment of good ties between older adults and their children through advocacy, sensitization and other measures. At the same time, provide more platforms for older adults to make friends, thereby promoting the relational integration.

## Data availability statement

Publicly available datasets were analyzed in this study. This data can be found at: https://charls.charlsdata.com/pages/Data/harmonized_charls/zh-cn.html.

## Author contributions

AM: Formal analysis, Methodology, Visualization, Writing – original draft. YC: Data curation, Methodology, Writing – review & editing. XT: Writing – review & editing QR: Writing – review & editing XR: Conceptualization, Supervision, Writing – review & editing.
