# Supplementary material for: Association of social integration with cognitive function trajectories among Chinese older adults: evidence from the China health and retirement longitudinal study (CHARLS)
Source: Front Aging Neurosci. 2024 Jan 11;15:1322974. doi: 10.3389/fnagi.2023.1322974 (PMC10808469; doi:10.3389/fnagi.2023.1322974)
Supplement: Supplementary file 1 [file Data_Sheet_1.docx]

Supplementary Material

# 1 Supplementary Figures and Tables

## Supplementary Figures

A total of 8723 older adults aged 60 years or older were surveyed in CHARLS 2013, of which a total of 6521 older adults (74.76%) completed the three periods of follow-up. Excluding those with more than two periods of missing data on cognitive function (n=2519) and missing variables (n=25), a total of 3977 older adults were finally included in the analysis. The sample screening process is shown in **Supplementary Figure 1.**

Participants aged 60 or above in CHARLS in 2013,N=8723

Excluded (N=2202)

1.Not followed up in 2015, N=1040

2.Not followed up in 2018, N=1162

Participants who completed two waves of follow-up, N=6521

Excluded (N=2544)

1.Measurements of cognitive function < 2 waves , N=2519

2.Missing variables, N=25

Participants in the final analysis, N=3977

Flow chart for the selection of the participants

**Supplementary Figure 1.** Flow chart shows the selection of the participants in this study.

## Supplementary tables

We have tried to shape the trajectory in different shapes. We fitted the trajectories in two steps, the first step to determine the number of trajectories and the second step to determine the morphology of the trajectories. In the first step to determine the number of groups, we first set all trajectories to 1 subterm, and then fitted models containing 2, 3, and 4 groups, respectively. The corresponding BICs of the models for groups 2 to 4 are -30283.76, -30078.69, and -30091.12, respectively. Based on the BIC values of the models, we determined the optimal number of groups to be 3. Then we tried different combinations of trajectories, and this step we considered additional model parameters including BIC, Entropy, AvePP and group membership statistically significant. The results for all models are presented in **Supplementary Table 1**.

**Supplementary Table 1.** Main parameters of different models.

| Model | Order^#^ | BIC | Entropy | Group membership and AvePP | | | | | |
| --- | --- | --- | --- | --- | --- | --- | --- | --- | --- |
|  |  |  |  | Group1(%) | AvePP | Group2(%) | AvePP | Group3(%) | AvePP |
| 1 | 2 2 2 | -30091.21 | 0.622 | 24.02* | 0.86 | 32.04* | 0.83 | 43.94* | 0.78 |
| 2 | 2 2 1 | -30087.06 | 0.622 | 43.94* | 0.78 | 24.12* | 0.86 | 32.04* | 0.83 |
| 3 | 2 2 0 | -30083.20 | 0.623 | 44.16* | 0.78 | 43.67* | 0.86 | 31.72* | 0.83 |
| 4 | 2 1 1 | -30082.85 | 0.622 | 24.02* | 0.86 | 43.94* | 0.78 | 32.04* | 0.83 |
| 5 | 2 1 0 | -30078.99 | 0.623 | 24.12* | 0.86 | 44.17* | 0.78 | 31.72* | 0.83 |
| 6 | 2 0 0 | -30138.58 | 0.605 | 29.94* | 0.88 | 42.27* | 0.76 | 27.79* | 0.81 |
| 7 | 1 1 1 | -30078.69 | 0.622 | 24.02* | 0.86 | 43.94* | 0.78 | 32.04* | 0.83 |
| **8** | **1 1 0** | **-30074.83** | **0.623** | **24.12*** | **0.86** | **44.17*** | **0.78** | **31.72*** | **0.83** |
| 9 | 1 0 0 | -30134.40 | 0.605 | 29.94* | 0.88 | 42.27* | 0.76 | 27.79* | 0.81 |
| 10 | 0 0 0 | -30520.80 | 0.602 | 17.64* | 0.81 | 44.72* | 0.79 | 37.64* | 0.84 |

^#^0=intercept, 1=linear, 2=quadratic； * indicates p<0.001

We added interaction terms for age, gender, marital status, household registration with relationship integration and community integration to the original regression model (five regressions were conducted), and the coefficients and significance of the interaction terms in the five regression models were shown in the **Supplementary Table 2.**

**Supplementary Table 2** Analysis of the interaction of relational integration and community integration with age, gender, marital status, education level and household registration

| **Model** | **Variable** | Low-decline group | |  | High-stable group | |
| --- | --- | --- | --- | --- | --- | --- |
|  |  | ***OR(95%CI)*** | ***P*** |  | ***OR(95%CI)*** | ***P*** |
| M_1_ | Relational*Age | 0.929(0.741,1.165) | 0.524 |  | 0.932(0.737,1.180) | 0.559 |
|  | Community*Age | 1.397(0.849,2.299) | 0.188 |  | 0.615(0.421,0.900) | 0.012^*^ |
|  |  |  |  |  |  |  |
| M_2_ | Relational*Sex | 0.980(0.796,1.208) | 0.853 |  | 1.067(0.879,1.295) | 0.510 |
|  | Community*Sex | 1.022(0.622,1.678) | 0.932 |  | 1.353(0.961,1.904) | 0.083 |
|  |  |  |  |  |  |  |
| M_3_ | Relational*Married | 1.072(0.800,1.437) | 0.641 |  | 1.162(0.856,1.578) | 0335 |
|  | Community*Married | 0.927(0.474,1.813) | 0.824 |  | 1.293(0.758,2.208) | 0.346 |
|  |  |  |  |  |  |  |
| M_4_ | Relational*Edu | 1.044(0.876,1.245) | 0.629 |  | 1.164(0.989,1.371) | 0.067 |
|  | Community*Edu | 0.908(0.597,1.380) | 0.651 |  | 0.793(0.602,1.045) | 0.099 |
|  |  |  |  |  |  |  |
| M_5_ | Relational*Household | 0.631(0.513,0.775) | ＜0.001^***^ |  | 1.459(1.272,1.674) | ＜0.001^***^ |
|  | Community*Household | 0.640(0.387,1.060) | 0.083 |  | 1.582(1.104,2.268) | 0.012^*^ |

OR: the odds ratio; CI : confidence interval; ^*^ indicates P < 0.05, ^**^ indicates P < 0.01, and ^***^ indicates P < 0.001;

We compared the differences in age, gender, marital status, and education level between the samples included in the analyses and the study participants who were excluded because they were missed or had missing values, in order to assess the impact of such differences on the subsequent analyses.The results were shown in **Supplementary Table 3**

**Supplementary Table 3** Analysis of lost/missing samples^#^

|  | valid sample（n=3977） | lost/missing （n=4746） | ***χ^2^*** | ***P*** |
| --- | --- | --- | --- | --- |
| Age |  |  | 515.898 | ＜0.001^***^ |
| 60~69 | 2984（55.05） | 2437（44.95） |  |  |
| ≥70 | 993（30.07） | 2309（69.93） |  |  |
| Sex |  |  | 254.629 | ＜0.001^***^ |
| Male | 2350（54.16） | 1989（45.84） |  |  |
| Female | 1627（37.13） | 2755（62.87） |  |  |
| Marital Status |  |  | 236.528 | ＜0.001^***^ |
| Married | 3425（49.88） | 3441（50.22） |  |  |
| Not married | 552（29.82） | 1299（70.18） |  |  |
| Educational level |  |  | 1.0e＋3 | ＜0.001^***^ |
| Illiterate | 553（20.99） | 2082（79.01） |  |  |
| Primary school | 1712（56.71） | 1307（43.29） |  |  |
| Junior high school and above | 938（64.60） | 514（35.40） |  |  |

^#^We did not include weights in this analysis.
